# Recurrent Lower Extremity Pseudocellulitis in a Liver Transplant Recipient: Suspected Tacrolimus-Associated Drug Eruption

**DOI:** 10.7759/cureus.116355

**Published:** 2026-09-16

**Authors:** Jennifer Acevedo, Damian Casadesus, Mickael B Birsingh, Emily Hartsell

**Affiliations:** 1 Internal Medicine, St. George's University School of Medicine, Miami, USA; 2 Internal Medicine, Jackson Memorial Hospital, Miami, USA; 3 Pathology, Jackson Memorial Hospital, Miami, USA

**Keywords:** cellulitis mimic, cutaneous adverse reaction, drug eruption, immunosuppression, liver transplantation, peripheral eosinophilia, pseudocellulitis, recurrent cellulitis-like eruption, skin biopsy, tacrolimus

## Abstract

Cutaneous drug eruptions can closely mimic cellulitis, creating a diagnostic challenge in transplant recipients receiving multiple potentially contributory medications. We report recurrent lower-extremity pseudocellulitis in a 55-year-old liver transplant recipient whose clinical course raised suspicion for a tacrolimus-associated drug eruption. Over 11 months, she was hospitalized 10 times and received multiple therapeutic and prophylactic antimicrobial regimens without sustained improvement. Repeated microbiologic studies, including bacterial, fungal, and acid-fast bacillus tissue cultures, were negative. Dermatologic evaluation and skin biopsy demonstrated a superficial and deep perivascular, perieccrine, and focally interstitial inflammatory infiltrate without evidence of infection or vasculitis. The relapsing course, negative microbiologic evaluation, and nonspecific inflammatory biopsy findings favored a noninfectious process. Tacrolimus was considered one possible contributor based on the reported onset after transplantation and recurrence during continued exposure, although uncertain latency and concomitant antimicrobial exposure limited causal attribution.

## Introduction

Tacrolimus is a calcineurin inhibitor widely used for maintenance immunosuppression after solid organ transplantation because it improves graft survival and reduces acute rejection [1]. Its established adverse effects include nephrotoxicity, neurotoxicity, hypertension, electrolyte abnormalities, and post-transplant diabetes mellitus [1-3]. Cutaneous reactions to systemic tacrolimus are uncommon and incompletely characterized, with reported presentations including eczematous eruptions and symmetrical drug-related intertriginous and flexural exanthema [2-5]. Because cutaneous drug eruptions may have nonspecific clinical and histopathologic findings, establishing a causal medication requires careful correlation with the clinical course and medication exposures [6-8].

Lower-extremity cellulitis may be difficult to distinguish from noninfectious conditions that cause similar erythema, edema, warmth, and tenderness. These conditions, collectively termed pseudocellulitis, include stasis dermatitis, lipodermatosclerosis, and lymphedema-associated inflammation [9-13]. Misdiagnosis may lead to repeated hospitalizations, unnecessary antimicrobial exposure, adverse effects, and increased healthcare utilization [9,14-16]. The distinction is particularly challenging in transplant recipients, in whom infection, medication-related reactions, and chronic venous or lymphatic disease may coexist.

We describe recurrent lower-extremity pseudocellulitis in a liver transplant recipient with chronic venous insufficiency and lymphedema who was receiving tacrolimus. A medication-related eruption, including a possible tacrolimus-associated reaction, remained under consideration after repeated antimicrobial treatment and negative microbiologic evaluation, although the available evidence did not establish definitive causality. This case illustrates the importance of considering infectious, medication-related, venous, and lymphatic etiologies when cellulitis-like eruptions repeatedly recur.

## Case presentation

A 55-year-old woman who underwent orthotopic liver transplantation in 2021 presented with recurrent painful, erythematous lower-extremity eruptions. Tacrolimus was initiated after transplantation for maintenance immunosuppression, and the patient reported that the eruptions began during the post-transplant period. However, the date of the first eruption and its precise interval from tacrolimus initiation could not be determined retrospectively. Approximately seven months before the index hospitalization, her tacrolimus dose was reduced from 1 mg twice daily to 1 mg every morning and 0.5 mg every evening. No formulation change was documented, and the eruptions continued after the dose reduction. Her other regularly documented medications included aspirin, losartan, nifedipine, rosuvastatin, magnesium oxide, and vitamin D supplementation. Her medical history included type 2 diabetes mellitus, hypertension, hyperlipidemia, obesity, osteoporosis, chronic bilateral lower-extremity lymphedema, chronic venous insufficiency treated with bilateral venous ablation, and a recent *Clostridioides difficile* infection treated with oral vancomycin.

During the 11 months preceding the index hospitalization, the eruptions became near-monthly and resulted in 10 hospitalizations. Although the episodes predominantly affected the right lower extremity during this period, they occurred on both lower extremities and did not consistently recur at identical sites. Each documented eruption resolved completely while tacrolimus therapy was continued, followed by recurrence during continued exposure. Given her comorbidities and chronic immunosuppression, the eruptions were initially treated as recurrent nonpurulent cellulitis. Therapeutic and prophylactic antimicrobial exposure included cephalexin, clindamycin, intravenous cefazolin, amoxicillin-clavulanate, and monthly intramuscular benzathine penicillin G. Complete records from all 10 hospitalizations were unavailable; therefore, the morphology, treatment, and precise antimicrobial exposure associated with every episode could not be reconstructed retrospectively.

**Figure 1:**
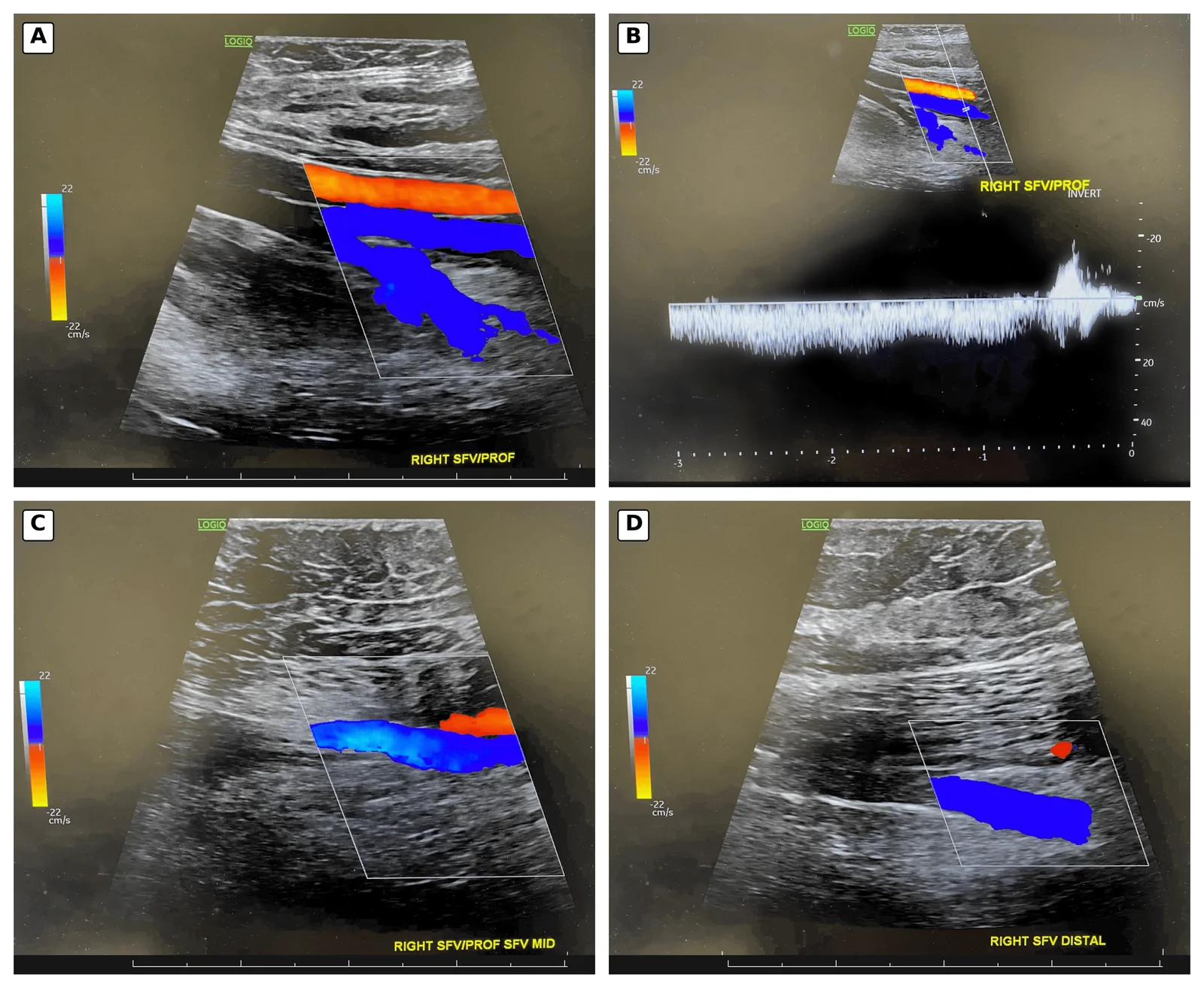
Right lower-extremity venous duplex ultrasonography Color Doppler images demonstrate flow within the right proximal femoral/profunda femoris veins (A), mid femoral vein (C), and distal femoral vein (D). Spectral Doppler demonstrates venous flow in the proximal femoral/profunda region (B). Overall findings were interpreted as showing no evidence of deep vein thrombosis.

**Figure 2:**
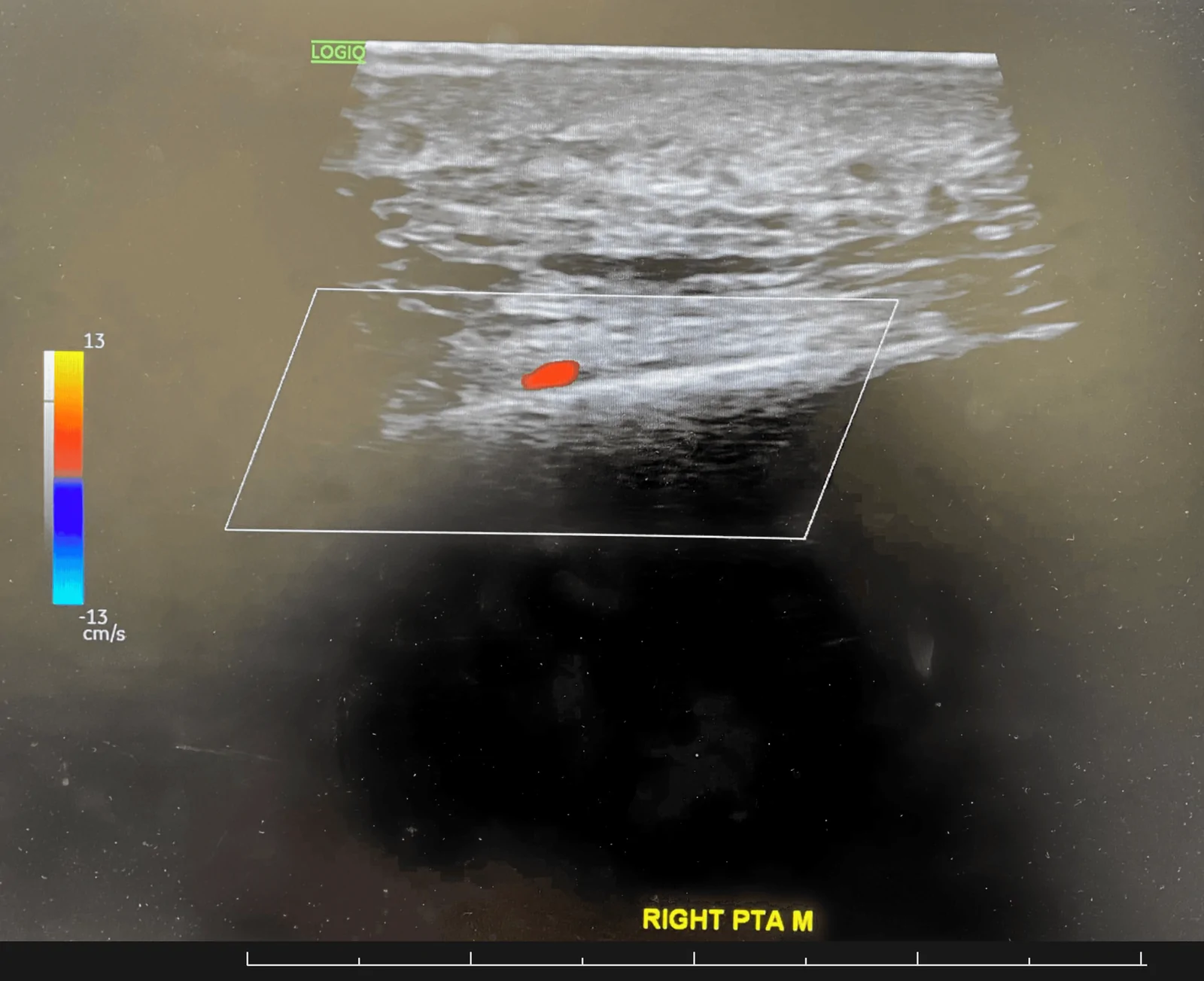
Subcutaneous edema visualized during right lower-extremity duplex ultrasonography The grayscale component demonstrates heterogeneous thickening and hypoechoic fluid tracking within the subcutaneous tissues, compatible with edema. No discrete drainable fluid collection was identified during the complete ultrasonographic examination.

One month before the index hospitalization, she presented with two days of worsening right lower-extremity pain and erythema accompanied by chills, nausea, and vomiting. She was afebrile and hemodynamically stable, with erythema and tenderness along the medial right thigh and calf. C-reactive protein was 5.8 mg/dL, and the erythrocyte sedimentation rate was 120 mm/hour; there was no leukocytosis. Duplex ultrasonography of the right lower extremity showed no deep venous thrombosis, and soft-tissue ultrasonography of the right calf demonstrated subcutaneous edema without a drainable collection (Figures 1, 2). Methicillin-resistant *Staphylococcus aureus* polymerase chain reaction testing was negative. She received empiric intravenous cefazolin followed by a five-day course of oral amoxicillin-clavulanate for presumed recurrent cellulitis. The eruption resolved completely while tacrolimus was continued, but another episode developed approximately one month later.

**Figure 3:**
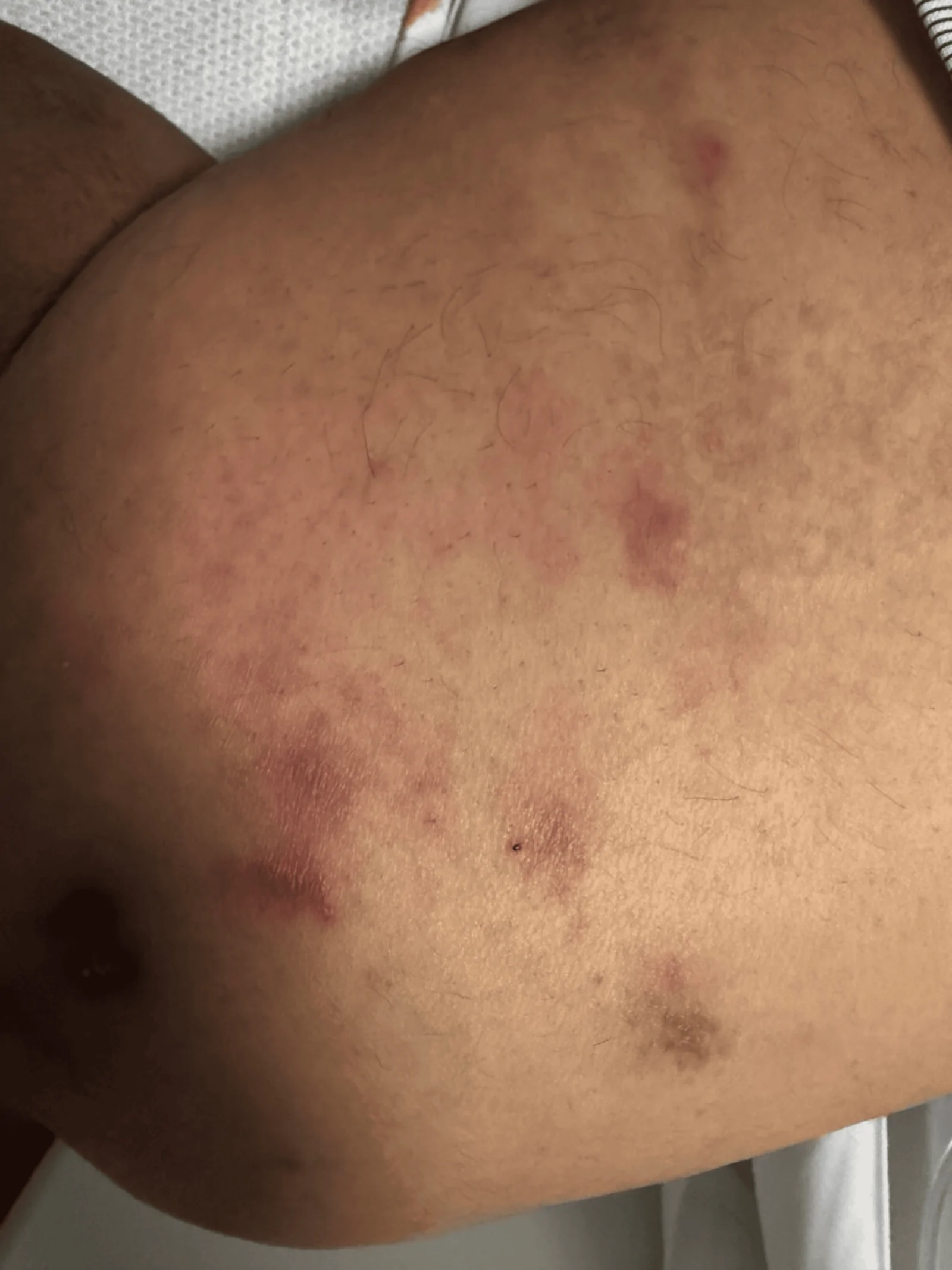
Clinical appearance of the recurrent posterior right thigh eruption

At the index hospitalization, she presented with recurrent painful lesions on the posterior right thigh. Examination revealed multiple discrete, well-demarcated erythematous plaques without fluctuance, purulent drainage, ulceration, or necrosis (Figure 3). She remained afebrile and had no leukocytosis, with a white blood cell count of 3.9 × 10⁹/L. C-reactive protein was 18.3 mg/dL, and the erythrocyte sedimentation rate was 120 mm/hour. Eosinophils accounted for 6.1% of leukocytes, corresponding to a calculated absolute eosinophil count of approximately 0.24 × 10⁹/L, which was within the normal range and did not represent absolute eosinophilia. Tacrolimus trough concentrations ranged from 4.8 to 5.7 ng/mL, compared with the transplant team’s patient-specific maintenance target of 2-5 ng/mL. These concentrations did not independently establish or exclude a drug eruption. Selected laboratory and microbiologic findings are summarized in Table 1.

**Table 1:** Summary of laboratory and microbiologic findings during the pre-index and index hospitalizations MRSA: Methicillin-resistant *Staphylococcus aureus*

Finding	Pre-index hospitalization	Index hospitalization	Reference/Target
White blood cell count	No leukocytosis	3.9 × 10⁹/L	3.4–9.6 × 10⁹/L
C-reactive protein	5.8 mg/dL	18.3 mg/dL	<0.5 mg/dL
Erythrocyte sedimentation rate	120 mm/hour	120 mm/hour	0–30 mm/hour (women ≥50 years)
Eosinophils	-	6.1%	0–5%
Calculated absolute eosinophil count	-	0.24 × 10⁹/L	0.03–0.48 × 10⁹/L
Tacrolimus trough concentration	-	4.8–5.7 ng/mL	2–5 ng/mL patient-specific target
MRSA polymerase chain reaction	Negative	-	Negative
Tissue cultures	-	Negative	Negative

**Figure 4:**
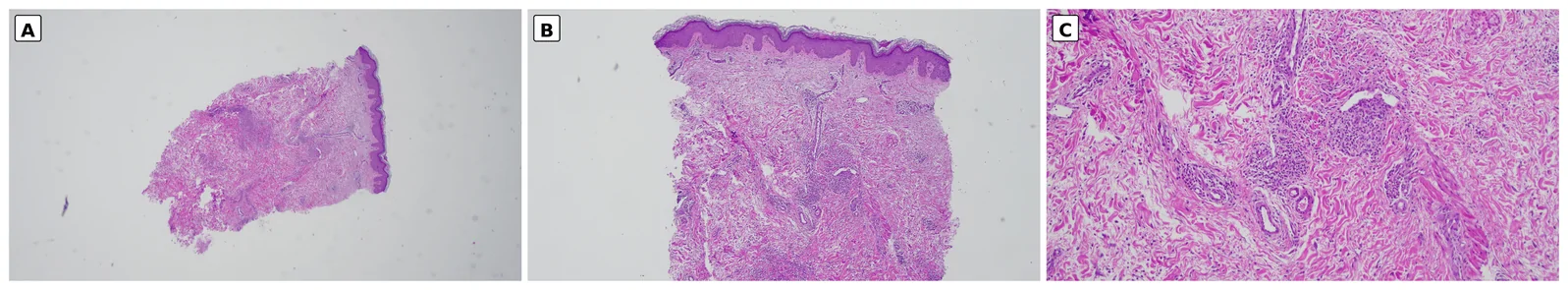
Histopathologic findings from the posterior left thigh punch biopsy Hematoxylin and eosin-stained sections are shown at low-power (A), intermediate-power (B), and high-power (C) views. Histopathologic examination demonstrated a superficial and deep perivascular, perieccrine, and focally interstitial inflammatory infiltrate composed of lymphocytes, neutrophils, and histiocytes, with mild dermal edema.

The prolonged relapsing course despite therapeutic and prophylactic antimicrobial therapy prompted reconsideration of the diagnosis. Dermatology expanded the differential diagnosis to include recurrent streptococcal cellulitis, stasis dermatitis, neutrophilic dermatosis, and a cutaneous drug eruption. A 3-mm punch biopsy of the posterior right thigh was obtained for histopathologic examination and bacterial, fungal, and acid-fast bacillus cultures, all of which were negative. Histopathologic examination demonstrated a superficial and deep perivascular, perieccrine, and focally interstitial infiltrate composed of lymphocytes, neutrophils, and histiocytes with mild dermal edema (Figure 4). Gram and periodic acid-Schiff stains showed no organisms, and there was no evidence of leukocytoclastic vasculitis or histologic features of chronic venous insufficiency. These findings favored a noninfectious inflammatory process but were not specific for a drug eruption.

Tacrolimus was considered a possible culprit because the eruptions reportedly began after its initiation and recurred during continued exposure. However, the precise latency was unknown, and concomitant antibiotics could not be excluded because their temporal relationships to individual episodes were incompletely documented. Tacrolimus was continued at the recommendation of the transplant hepatology team. The available records did not document a confirmed rejection episode, although subsequent records noted persistent liver enzyme elevations that prompted evaluation for possible chronic rejection. Bilirubin levels remained preserved, and hepatic imaging demonstrated patent vasculature without focal lesions or fluid collections. The records did not document a formal assessment of alternative immunosuppressive regimens or specific reasons that substitution was considered unsuitable. Therefore, neither dechallenge nor rechallenge could be assessed.

Eight weeks after the index hospitalization, the patient was readmitted with a similar painful eruption predominantly involving the contralateral right lower extremity. Dermatologic examination demonstrated dark-brown patches and indurated plaques on both lower extremities, with superficial ulcerations and greater edema on the right. Her ALT-70 (asymmetry, leukocytosis, tachycardia, age ≥70 years) score was 0 [17]: bilateral involvement contributed no points for asymmetry, and she received no additional points because she had no leukocytosis, her heart rate was 86 beats/min, and she was younger than 70 years. Dermatology favored pseudocellulitis associated with chronic venous insufficiency, lipodermatosclerosis, and lymphedema. Although the ALT-70 score supported a noninfectious rather than bacterial process, it did not identify a specific etiology. Nevertheless, recurrence after complete resolution, prior negative microbiologic studies, and biopsy findings compatible with a drug eruption supported continued consideration of a medication-related cause, including tacrolimus.

## Discussion

This case describes an uncommon pseudocellulitis-like presentation in which tacrolimus was considered a possible cause of a recurrent cutaneous eruption in a liver transplant recipient. Systemic cutaneous reactions to tacrolimus are rarely reported despite its widespread use after transplantation. Published phenotypes include eczematous eruptions and symmetrical drug-related intertriginous and flexural exanthema, underscoring the absence of a single defining morphology [4,5]. Improvement after tacrolimus withdrawal or substitution has supported causality in prior reports [4,5]. In this patient, tacrolimus was continued at the recommendation of the transplant hepatology team. Although subsequent records documented evaluation for possible chronic rejection, the available records did not document a formal assessment of alternative immunosuppressive regimens or specific reasons that tacrolimus substitution was considered unsuitable. Consequently, causality could not be evaluated through dechallenge or rechallenge.

Recurrent bacterial cellulitis was initially plausible given the patient's lymphedema, venous insufficiency, obesity, diabetes mellitus, and immunosuppression. Over time, however, the stereotyped recurrences, lack of sustained response to therapeutic and prophylactic antibiotics, absence of fever or persistent leukocytosis, and negative tissue cultures favored a noninfectious mimic. This distinction is clinically important because approximately one-third of patients hospitalized with presumed lower-extremity cellulitis ultimately receive an alternative diagnosis [9-13]. Early dermatology consultation improves diagnostic accuracy and can reduce unnecessary antibiotic exposure and healthcare utilization [14-16].

Biopsy supported a noninfectious inflammatory process while helping exclude competing diagnoses. Histopathologic examination demonstrated a superficial and deep perivascular, perieccrine, and focally interstitial infiltrate composed of lymphocytes, neutrophils, and histiocytes with mild dermal edema; no organisms, leukocytoclastic vasculitis, or features of chronic venous insufficiency were identified. Although nonspecific, this pattern falls within the histopathologic spectrum of delayed cutaneous drug eruptions [6,7]. Weyers and Metze reported that eosinophils and/or neutrophils are present in most drug eruptions and noted that neutrophilic infiltrates, although less common, may have greater diagnostic significance than eosinophilic infiltrates [8]. The biopsy demonstrated tissue neutrophils, but peripheral eosinophils accounted for 6.1% of leukocytes, corresponding to a calculated absolute eosinophil count of approximately 0.24 × 10⁹/L. Although the histopathologic pattern was compatible with a drug eruption, it was nonspecific and required correlation with the clinical course, medication exposures, and microbiologic findings.

Tacrolimus remained one potential culprit because the patient reported that the eruptions began after its initiation and recurred during continued exposure. However, cephalexin, clindamycin, cefazolin, amoxicillin-clavulanate, and benzathine penicillin G were also potential causes, while chronic venous and lymphatic disease provided additional competing explanations. Complete medication records from all 10 hospitalizations were unavailable; therefore, the timing of each antimicrobial exposure relative to individual episodes could not be reconstructed. The item-by-item Naranjo assessment yielded a score of +3, classifying the association with tacrolimus as possible but neither establishing causality nor resolving the competing explanations (Table 2) [18]. The unknown latency from tacrolimus initiation, absence of dechallenge or rechallenge, and lack of a demonstrated dose-response relationship further limited causal attribution. Follow-up beyond the subsequent readmission was unavailable. Overall, the clinical, microbiologic, and histopathologic findings supported a recurrent noninfectious inflammatory eruption and demonstrated the value of multidisciplinary evaluation when cellulitis-like lesions recur despite antimicrobial therapy.

**Table 2:** Item-by-item Naranjo Adverse Drug Reaction Probability Scale assessment for tacrolimus [18]

Naranjo item	Assessment	Score	Case-specific rationale
1. Previous conclusive reports	Yes	1	Systemic tacrolimus-associated cutaneous reactions have been reported.
2. Event after drug administration	Yes, with uncertain latency	2	The patient reported that the eruptions began after tacrolimus initiation, but the precise interval was unavailable.
3. Improvement after withdrawal	Not assessed	0	Tacrolimus was not withdrawn.
4. Recurrence after rechallenge	Not assessed	0	No tacrolimus rechallenge occurred.
5. Alternative causes	Yes	-1	Concomitant antibiotics and chronic venous and lymphatic disease were plausible competing explanations.
6. Reaction after placebo	Not assessed	0	No placebo was administered.
7. Toxic drug concentration	No	0	Trough concentrations were 4.8–5.7 ng/mL against a patient-specific target of 2–5 ng/mL and were not independently diagnostic.
8. Dose-response relationship	Not demonstrated	0	The eruptions continued after dose reduction, and no clear relationship between dose and severity was documented.
9. Similar reaction with previous exposure	Not documented	0	No similar reaction during a separate previous exposure to tacrolimus or a related agent was documented.
10. Objective evidence	Yes	1	The eruption was documented clinically and histopathologically, although the biopsy findings were nonspecific.
Total score		3	Possible adverse drug reaction

## Conclusions

Tacrolimus-associated drug eruption should be considered in transplant recipients with recurrent cellulitis-like eruptions that do not respond durably to appropriate antimicrobial therapy. In this patient, the onset of eruptions after transplantation, recurrence during continued tacrolimus exposure, lack of sustained response to therapeutic and prophylactic antimicrobials, negative microbiologic evaluation, and compatible inflammatory biopsy findings supported a possible association with tacrolimus. Definitive causality could not be established because the precise exposure timeline was unavailable, the histopathologic findings were nonspecific, concomitant antimicrobials could not be excluded, and neither dechallenge nor rechallenge was performed. Chronic venous insufficiency, lipodermatosclerosis, and lymphedema also remained plausible contributors to the pseudocellulitis presentation. This case highlights the importance of considering medication-related eruptions alongside venous, lymphatic, and infectious etiologies when cellulitis-like lesions recur despite appropriate treatment.
